# Volumetric Analyses of Dysmorphic Maxillofacial Structures Using 3D Surface-Based Approaches: A Scoping Review

**DOI:** 10.3390/jcm13164740

**Published:** 2024-08-12

**Authors:** Annalisa Cappella, Francesca Gaffuri, Josh Yang, Francesco Carlo Tartaglia, Riccardo Solazzo, Francesco Inchingolo, Gianluca Martino Tartaglia, Chiarella Sforza

**Affiliations:** 1 U.O. Laboratory of Applied Morphology, IRCCS Policlinico San Donato, 20097 San Donato Milanese, Italy; annalisa.cappella@unimi.it; 2 Department of Biomedical Sciences for Health, University of Milan, 20133 Milan, Italy; 3 Department of Biomedical, Surgical and Dental Sciences, University of Milan, 20122 Milan, Italy; francesca.gaffuri@unimi.it; 4 Fondazione IRCCS Cà Granda, Ospedale Maggiore Policlinico, 20122 Milan, Italy; 5 Harvard School of Dental Medicine (HSDM), Harvard University, Boston, MA 02115, USA; josh_yang@hsdm.harvard.edu; 6 Department of Biomedical Sciences, Humanitas University, 20072 Pieve Emanuele, Italy; francesco.tartaglia@st.hunimed.eu; 7 LAFAS (Laboratory of Functional Anatomy of the Stomatognathic System), Department of Biomedical Sciences for Health, University of Milan, 20133 Milan, Italy; chiarella.sforza@unimi.it; 8 Department of Interdisciplinary Medicine, University of Bari “Aldo Moro”, 70124 Bari, Italy; francesco.inchingolo@uniba.it

**Keywords:** imaging, three-dimensional, optical imaging, anthropometry, facial dysmorphology, cleft lip and palate, syndromic patients, 3D volumetric methods, dentistry, cranio-facial anatomy

## Abstract

**Background/Objectives**: Three-dimensional (3D) analysis of maxillofacial structures in dysmorphic patients offers clinical advantages over 2D analysis due to its high accuracy and precision in measuring many morphological parameters. Currently, no reliable gold standard exists for calculating 3D volumetric measurements of maxillofacial structures when captured by 3D surface imaging techniques. The aim of this scoping review is to provide an overview of the scientific literature related to 3D surface imaging methods used for volumetric analysis of the dysmorphic maxillofacial structures of patients affected by CL/P or other syndromes and to provide an update on the existing protocols, methods, and, when available, reference data. **Methods**: A total of 17 papers selected according to strict inclusion and exclusion criteria were reviewed for the qualitative analysis out of more than 4500 articles published between 2002 and 2024 that were retrieved from the main electronic scientific databases according to the PRISMA-ScR guidelines. A qualitative synthesis of the protocols used for the selection of the anatomical areas of interest and details on the methods used for the calculation of their volume was completed. **Results**: The results suggest a great degree of heterogeneity between the reviewed studies in all the aspects analysed (patient population, anatomical structure, area selection, and volume calculation), which prevents any chance of direct comparison between the reported volumetric data. **Conclusions**: Our qualitative analysis revealed dissimilarities in the procedures specified in the studies, highlighting the need to develop uniform methods and protocols and the need for comparative studies to verify the validity of methods in order to achieve high levels of scientific evidence, homogeneity of volumetric data, and clinical consensus on the methods to use for 3D volumetric surface-based analysis.

## 1. Introduction

Maxillofacial dysmorphisms include congenital or acquired [1,2] malformations, disruptions, deformations, and dysplasia [3,4] and significantly impact the lives of patients [5,6]. Functional impairments may affect breathing, speech, feeding, and vision abilities [7], thus requiring long-term treatments performed by interdisciplinary teams [8,9,10] aimed at restoring the morphology and the functionality of the affected structures [11].

Three-dimensional (3D) technologies [12,13,14,15] have revolutionised the management of maxillofacial dysmorphisms [15,16]. The use of digital 3D models of maxillofacial structures facilitates clinical and diagnostic assessments, virtual surgical planning, and surgical procedures [12,17], enabling detailed qualitative and quantitative evaluations. Techniques for 3D surface imaging such as stereophotogrammetry and laser scanning are the gold standard for 3D analysis of the face [18], as they are fast, safe (radiation-free), and non-invasive [19,20]. Their use has introduced advanced surface-based methods and related measurements to the facial anthropometric analyses of healthy subjects and patients with maxillofacial dysmorphisms [21,22,23,24] as opposed to the simpler linear and angular measurements implemented by conventional anthropometry [25,26]. Despite the advances in 3D technologies, gaps still remain in the existing literature regarding volumetric analysis of maxillofacial soft tissues, particularly in patients with maxillofacial dysmorphisms. Nevertheless, volumetric data could provide more comprehensive insights than unidimensional or bidimensional measurements and are becoming increasingly important in the clinical evaluation and treatment planning of patients. Honrado and Larrabee [16] already understood the potential of 3D technologies to accurately measure soft tissue volumetric changes in patients undergoing facial and orthognathic procedures; more recently, other authors [27,28] have highlighted the great importance of 3D volumetric analyses in the evaluation of patients affected by CL/P, as they allow for the assessment of the bone deficiency at the level of the cleft, the monitoring of maxillofacial development, and the follow-up of rehabilitative and surgical outcomes. Finally, due to the soft tissue paradigm [29,30], the field of plastic and aesthetic surgery has recently experienced a vivid change in the planning of surgical interventions to achieve a balanced face in terms of harmony, volume, and symmetry, through the 3D volumetric analysis of maxillofacial structures [31]. However, the lack of consensus on the protocols and methods used for the 3D volumetric analysis of dysmorphic maxillofacial structures is a cause for concern. The methods implemented may depend on the type of anatomical structure to be analysed and the software and technology used, and each protocol may also differ in terms of accuracy and reliability. In other words, different methodologies may not provide equivalent measurements.

This scoping review aims to provide an updated overview of the available protocols for the three-dimensional volumetric analysis of dysmorphic maxillofacial structures acquired using optical systems. It attempts to open a discussion on the importance of reference data and to guide researchers towards the development of validated protocols that would allow volumetric data to be compared in clinical settings. After an initial examination of the available literature, the results of the selected articles appeared to be highly variable and inconsistent. Therefore, a scoping review was deemed appropriate for the purposes of this study.

## 2. Materials and Methods

This scoping review was conducted in accordance with the recommendations of the Preferred Reporting Items for Systematic reviews and Meta-Analyses extension for Scoping Reviews (PRISMA-ScR) guidelines [32]. The question for this scoping review, formulated according to the PCC (Participants, Concept, and Context) framework, was “What are the protocols and associated data used to measure the volume (Concept) of facial structures in dysmorphic subjects (Participants) when acquired using 3D surface imaging techniques (Context)?”.

### 2.1. Search Strategy

A detailed search of articles published in the literature from 2002 to 30 June 2024 was carried out on 3 July 2024 in the databases Scopus, Embase, PubMed, and Web of Science using the search terms as listed in Table 1. Reference checking and citation tracking were performed to identify additional records. No grey literature was included in the search strategy. No restrictions were applied according to the type of publication, but only English language publications were considered.

### 2.2. Eligibility and Exclusion Criteria

The eligibility criteria for the study selection were as follows: studies focusing on human patients affected by maxillofacial dysmorphologies or syndromes; studies using optical surface-based methods for the reproduction of 3D models of the scanned surface and calculation of volume; and studies providing a quantitative volumetric assessment of the maxillofacial structures.

Studies were excluded if they focused on healthy subjects without syndromic dysmorphism or malformation of the maxillofacial region, analysed 3D maxillofacial models scanned by non-surface optical methods (such as CT, CBCT, radiography, or MRI), or reported data other than volume. Finally, studies on surgical interventions were only included if they also provided data on the preoperative period.

### 2.3. Study Selection

The studies retrieved from the electronic search, reference checking, and citation tracking were imported and initially screened to exclude duplicates in the web application Rayyan (Qatar Computing Research Institute, Doha, Qatar) [33]. After removing duplicate records, the initial study selection based on title and abstract was performed by two assessors (F.G. and J.Y.) who independently screened the papers against inclusion and exclusion criteria. Disagreements between the two assessors were resolved by a third assessor (A.C.).

### 2.4. Data Charting

Data collection from the selected studies was carried out using a standardised Excel data sheet (Microsoft Office Excel, 2019). The data-charting Excel form was initially drafted by one reviewer (J.Y.) and then adapted through iterative discussion with the other two reviewers (F.G. and A.C.) to determine the variables to be extracted from each selected study and thus the relevant data to be included in this review.

### 2.5. Data Items

For each article selected, the following data were reported: the type and purpose of the study; the pathology affecting the patients analysed; the age group, sex, and ethnicity of the patients; sample size; scanning system type; the name and manufacturer of the scanning system; the software used for the volumetric analysis; the anatomical structure analysed and the method used to define the region of interest; the description of the protocol used to calculate the volume; whether the protocol was validated; and whether intra- and inter-operator reliability were verified.

### 2.6. Critical Appraisal Assessment

The primary aim of this scoping review was to map and report existing methods, protocols, and available data for the volumetric measurements of maxillofacial structures in dysmorphic patients rather than to evaluate individual study results and provide a clinically meaningful answer to a question. Therefore, in accordance with the PRISMA extension for scoping reviews (PRISMA-ScR) [32,34], a critical appraisal of the included studies (risk of bias assessment) was not performed.

### 2.7. Synthesis of Results

The results were synthesised in a narrative format using summarising tables and figures. Generalisations of study data and patients’ characteristics were reported. Details on the selection of the area of interest (the anatomical structure analysed) and the method of volume calculation were reported with studies grouped according to the protocols used for each of the two steps. Finally, descriptive statistics were used to summarise the results of the studies reporting the volumetric data of dysmorphic maxillofacial structures, grouping the studies according to the type of reporting volumetric data: actual volume, volume changes, and dimensionless volumetric indices.

## 3. Results

**Figure 1:**
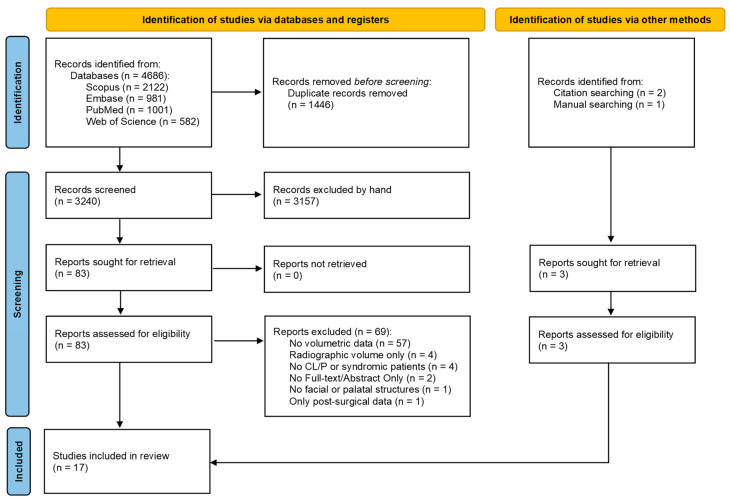
Preferred Reporting Items for Systematic Review and Meta-Analysis (PRISMA) flow diagram.

A total of 4686 articles were initially identified from four databases: Scopus, Embase, PubMed, and Web of Science. After removing duplicate studies (n = 1446) and screening for titles and abstracts, 3157 articles were excluded as inappropriate studies, while the full texts of 83 articles were selected for an in-depth review. Of these, 69 publications were excluded based on the eligibility criteria. Reasons for exclusion after the full-text reading included evaluation of parameters other than volume, absence of dysmorphic or syndromic patients, use of technologies other than 3D surface optical devices, and examination of anatomical structures other than maxillofacial ones. From the remaining 14 selected articles that met the selection criteria, three additional articles were retrieved through a manual search, resulting in 17 articles being included in this scoping review [27,35,36,37,38,39,40,41,42,43,44,45,46,47,48,49,50]. The PRISMA flow diagram is shown in Figure 1.

### 3.1. Overview of Studies

The selection yielded 17 studies that measured the volumes of maxillofacial structures in dysmorphic or syndromic patients using 3D surface optical acquisition methods. In total, seven studies were conducted in Italy [35,36,37,40,45,46,47], four in the United States (U.S.) [41,43,48,50], two in Brazil [27,38], one in India [39], one in Turkey [44], one in the Netherlands [49], and one in China [42].

#### Individual Study Characteristics

**Figure 2:**
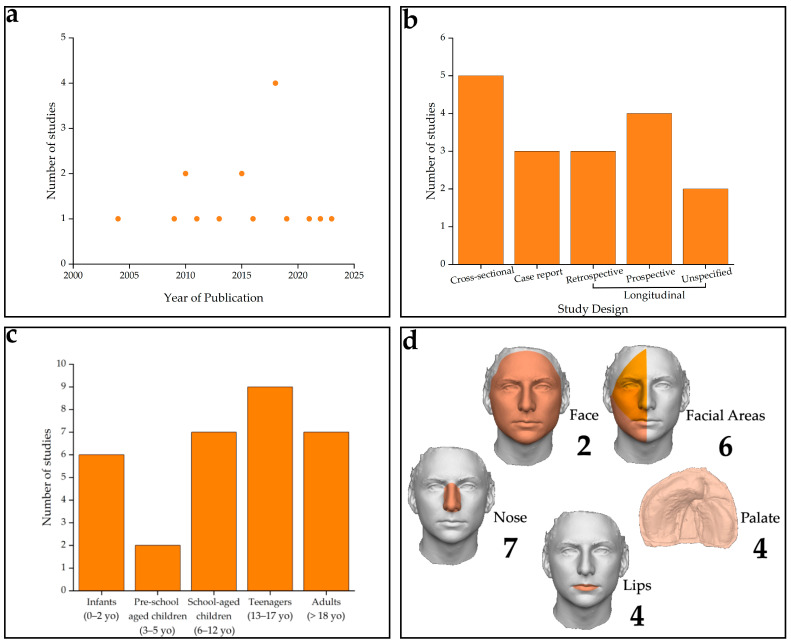
Individual Study Characteristics: (**a**) number of studies published per year; (**b**) study design; (**c**) age groups analysed across individual studies; (**d**) analysed structures in individual studies.

Individual study characteristics are depicted in Figure 2. Overall, the included studies were published between 2004 and 2023, showing a constant interest in the topic with a peak in 2018. Concerning the study design, all of the studies were observational and clinical, with 29.4% of the studies being identified as cross-sectional [35,36,37,40,44], 17.7% as case reports [42,46,50], and 52.9% as longitudinal, of which 17.7% were retrospective [41,48,49], 23.4% were prospective [38,39,43,47], and 11.8% were unspecified [27,45].

The studies comprehensively calculated volume measurements of the face or other maxillofacial structures (i.e., facial areas, lips, nose, and palate) in a total of 476 patients, including 328 patients with CL/P and 148 syndromic patients. While only two studies analysed the whole face [36,41], the majority focused on other maxillofacial structures. Six studies [36,42,44,46,48,50] analysed large areas of the face, seven studies [35,36,37,39,43,44,49] analysed the nose, four studies [35,37,44,47] considered the lips, and the remaining four [27,38,40,45] analysed the palate. Four studies analysed [35,36,37,44] more than one of the above maxillofacial structures, with the nose and lips being measured the most in the same study. Only one study [36] evaluated the nose together with the whole face and wider facial areas.

Age groups were classified as children (including infants [0–2 years old], pre-school children [3–6 years], school-aged children [7–12 years]), teenagers (13–18 years), and adults (18+ years), as reported in Table 2. While most studies (65%) specified the age of their participants [27,38,39,40,41,42,43,45,46,47,50], the remaining studies [35,36,37,44,48,49] heterogeneously analysed subjects of multiple and different ages without specifying the numbers for each specific age or age class. In the studies including only subadult participants (children and/or teenagers), seven studies [27,38,39,40,43,45,47] focused only on children under 13 years (269 patients, 57% of the total patients), two case-reports [46,50] analysed teenagers (a total of two patients), and one study [41] analysed both children and teenagers (a total of 12 patients). Finally, one case report focused exclusively on one adult [42], while six studies [35,36,37,44,48,49] evaluated both subadults and adults, analysing a total of 192 participants, without reporting the number per specific age group.

Considering the pathologies affecting the patients analysed, as summarised in Table 3, most studies examined CL/P patients [27,38,39,43,44,45,47,48,49,50], while seven focused on syndromic subjects affected by hemifacial microsomia [42], syndromic craniosynostosis (Crouzon, Apert, Pfeiffer, Saethre–Chotzen, and unknown syndromes) [41], and Parry–Romberg [46], Down [35,37], Marfan [40], and Moebius [36] syndromes.

The final individual study characteristics analysed related to the objectives and results of the studies, which were classified as either surgical or morphological. Studies in which measurements were primarily calculated to aid in surgical planning or to assess volumetric changes after surgery were defined as “surgical”, whereas those in which volumetric measurements were reported as descriptors to be used and compared in the clinical setting were defined as “morphological”. Approximately 71% of the studies (12 out of 17) [27,38,39,41,42,43,45,46,47,48,49,50] focused on the assessment of surgical outcomes, specifically changes in volume and/or other linear and surface parameters related to a surgical or orthopaedic intervention, while only the remaining 29% (5 of 17) [35,36,37,40,44] were diagnostic or descriptive studies aimed at characterizing maxillofacial phenotypes and morphological features in patients with maxillofacial dysmorphisms or syndromes, often in comparison to healthy subjects. Among the studies with surgical intent, two studies had additional purposes: one [45] aimed to develop a method to estimate the volume of the dental arches in CL/P patients, and the other [43] aimed to propose an automated objective protocol to measure 3D digital nasal models of UCL patients with nasal deformities.

Additional information concerning the patients from each singular study, such as the sample size, percentage of females, age groups included, mean age and/or age range, and ethnicity, is summarised in Table 4.

### 3.2. Summary of the Methodologies Employed in the Selected Studies

**Figure 3:**
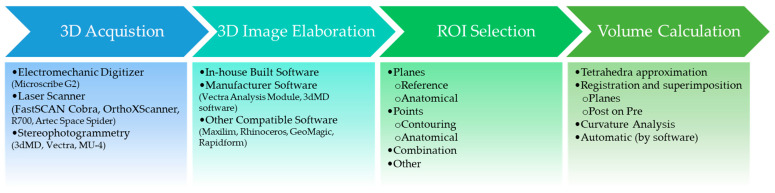
Workflow summarising the methodological steps from scanning to volume calculation.

All of the studies included in this review assessed the volume of various dysmorphic maxillofacial features of patients with CL/P or other syndromes. The assessment of volume was detailed in most of the studies (14 out of 17) and involved several steps, as shown in Figure 3. In general, the first step consisted of the 3D reproduction of the anatomical structure, which was obtained directly from the subjects [35,36,37,39,41,42,43,44,46,47,48,49,50] or indirectly from casts [27,38,40,45], using different 3D technologies: electromechanical digitizers, laser scanners, and stereophotogrammetry. Once the 3D image depicting the face or another anatomical structure (ROI, region of interest) was obtained, the ROI needed to be selected by using landmarks, planes, or both in different 3D image elaboration software. The volume calculation could then be performed three-dimensionally, regardless of which of the various proposed methods was used to select the ROI.

### 3.3. Image Acquisition and Software

The 3D technology used to acquire the facial surfaces and the software used for the 3D analysis are summarized in Table 5. Ten studies used stereophotogrammetry [41,42,43,44,45,46,47,48,49,50], five used a laser scanning system [27,37,38,39,40], and two employed electromechanical digitizers [35,36].

#### 3.3.1. Stereophotogrammetry

The majority of the studies used a stereophotogrammetry system. Seven used a device from 3dMD (Atlanta, GA, USA), specifically the systems 3dMD face [42,44,49,50], 3dMD cranial [43], 3dMD trio [47], and MU-4 [41], while the others used devices from Canfield Scientific Inc. (Fairfield, NJ, USA), specifically the systems Vectra M3 [45,46] and Vectra XT [48]. Among the studies using 3dMD systems, in three studies, the volumetric analysis was performed using the company’s software (3dMDVultus, 3dMD, Atlanta, GA, USA) [41,42,44], in one study it was performed using 3dMDVultus in combination with GeoMagic Wrap (Artec 3D, Senningerberg, Luxembourg) [47], and in the other three studies, it was performed using an alternative software such as Maxilim version 2.2.2.1 (Medicim NV, Mechelen, Belgium) in combination with the manufacturer’s software 3dMDpatient version 3.0.1 (3dMD, Atlanta, GA, USA) [49], Rapidform 2006 (INUS Technology Inc., Seoul, Republic of Korea) associated with InVivo version 5.2.3 (Anatomage Inc., Santa Clara, CA, USA) [50], or an in-house software developed by the authors [43]. Meanwhile, all three studies employing Vectra imaging systems used the provided manufacturer software (Mirror Imaging Software, Canfield Scientific Inc., Fairfield, NJ, USA) [45,46,48]. Maxillofacial structures analysed in these studies included the whole face [42], the nose [43,44,48], the maxilla [50], the chin [44], the upper lip [47], and the palate [45]. The maxillofacial structures were acquired directly from the patients in all but one study, which used palatal casts [45].

#### 3.3.2. Laser Scanning

Five studies used a laser scanning system: 3Shape’s R700™ scanner (3Shape, Copenhagen, Denmark) [27,38], FastSCAN™ COBRA C1™ (Polhemus, Colchester, CT, USA) [37], orthoX^®^ scanner (OrthoXscan, Dentaurum GmbH&co, Ispringen, Germany) [40], and 3D Artec Space Spider (Artec 3D, Senningerberg, Luxembourg) [39]. The software used for the 3D volumetric analysis included Mirror Imaging Software (Canfield Scientific Inc, Fairfield, NJ, USA) [27,38], Rhinoceros Nurbs for Windows 4.0 (Robert McNeal, Seattle, WA, USA) [37], and GeoMagic Freeform Plus^®^ version V2017 (Artec 3D, Senningerberg, Luxembourg) [39], although one study did not specify which software was used [40]. The maxillofacial structures captured and analysed included the palate [27,38,40], the nasolabial area [37], and the nose [39]. In three studies, the 3D palatal scans were acquired indirectly from casts [27,38,40].

#### 3.3.3. Electromechanical Digitizers

Finally, two studies used electromechanical digitizers to obtain the 3D reproduction of the faces using landmarks [35,36]. The device used in both studies was the Microscribe G2 (Immersion Corporation, San Jose, CA, USA) and an in-house-built software, used to calculate the volume [35,36]. One study [35] assessed the volume of the nose and lips, while the other [36] calculated the volume of the whole face and other facial regions relevant to the forehead, maxilla, mandible, and nose.

### 3.4. Selection of the Structure of Interest

**Figure 4:**
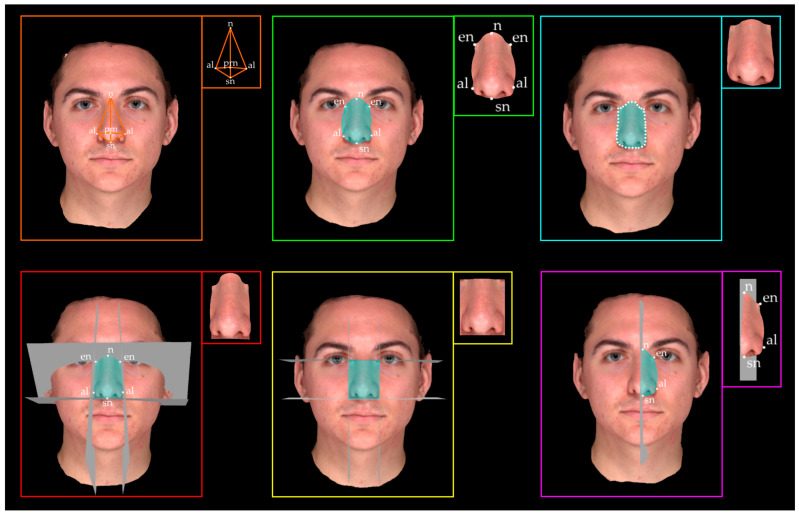
Summary of protocols for ROI selection and the resulting ROI. The ROI selection can occur via approximation with polyhedra (orange), anatomical (green) or contouring (light blue) landmarks, landmarks-based planes (red) or reference arbitrarily chosen planes (yellow), or by using a combination of both landmarks and planes (purple). Definition of anthropometric landmarks: n: nasion; en: endocanthion; al: alar; sn: subnasale; prn: pronasale.

The protocols used to select the ROI(s) differed between the included studies (Table 5 and Figure 4). Overall, regardless of the typology of the ROI, their definition can be based on landmarks, both anatomical and contouring (the latter arbitrarily chosen by the operator), landmarks-based or reference planes, or by using a combination of both. In one study, no ROI selection was performed because the volumetric assessment considered the entire face [41].

#### 3.4.1. Landmarks-Based Methods

Landmarks-based methods involve the selection of the ROI by means of either anatomical/anthropometric landmarks or contour points that outline the structure.

Four studies [35,37,46,47] selected the dysmorphic facial structure(s) of interest using only anatomical landmarks and two different approaches. Ferrario et al. [35] and Sforza et al. [37] used the digitised anatomical landmarks to reconstruct polyhedra (as tetrahedra) that could geometrically approximate the structure of interest (Figure 4, orange panel); Pucciarelli et al. [46] and Rizzo et al. [47] exploited the ability of the software used to automatically select the area enclosed by the digitised landmarks (Figure 4, green panel). Susarla et al. [48] used anatomical landmarks as a reference to identify the structure of interest and then manually selected the area enclosed by the contouring landmarks (Figure 4, green panel).

In contrast to the use of anatomical landmarks, three studies [27,38,45] used contouring landmarks to select the structure of interest. All of the studies using arbitrary landmarks analysed palatal structures (Figure 4, light blue).

#### 3.4.2. Planes-Based Methods

Four studies [39,40,44,49] used planes to select the ROIs. Van Loon et al. [49] and Chattopadhyay et al. [39] positioned one or more vertical and/or horizontal planes passing through anatomical landmarks to delineate the structures (Figure 4, red panel), while no landmarks were used to select the ROIs. In contrast, Ozdemir et al. [44] used horizontal and vertical reference planes that did not pass through specific anatomical landmarks (Figure 4, yellow panel). Paoloni et al. [40] explicitly stated that “a gingival plane and a distal plane were used as the boundaries of the palate. The gingival plane was obtained by connecting the centre of the dentogingival junction of all erupted permanent and deciduous teeth. The distal plane was created by two points on the distal margin of the second deciduous molars perpendicular to the gingival plane.” Thus, they used anthropometric landmarks through which planes could pass to delineate the ROI.

#### 3.4.3. Combination: Landmarks- and Planes-Based Methods

In one study [36], ROI selection was performed using both landmarks and planes. Sforza et al. [36] used a horizontal plane passing through anatomical landmarks to delineate the posterior boundary of the facial structures. This approach was applied in particular to calculate the volume of large facial areas such as the forehead, the maxilla, and the mandible, while anthropometric landmarks of the nose and lips were used to geometrically approximate the structures of interest as polyhedra, similar to prior studies [35,37] (Figure 4, purple panel).

#### 3.4.4. Other Methods

The remaining three studies [42,43,50] did not provide detailed information on ROIs selection. One study [50] performed a “manual” selection following the editing functions of the software used for the analysis. Another [42] created a “template” facial area on which post-surgical 3D images were subsequently superimposed to assess the volumetric changes. Finally, one study [43] used an automated method based on contour and curvature analysis, allowing for the detection of two local minima of the curvature angle at the nose corners, the global maximum of the curve at the nasal dorsum, and the intersection of the contour with the mid-facial plane as a reference, thus identifying the anatomical limits of the nose.

### 3.5. Protocols for Volume Calculation

**Figure 5:**
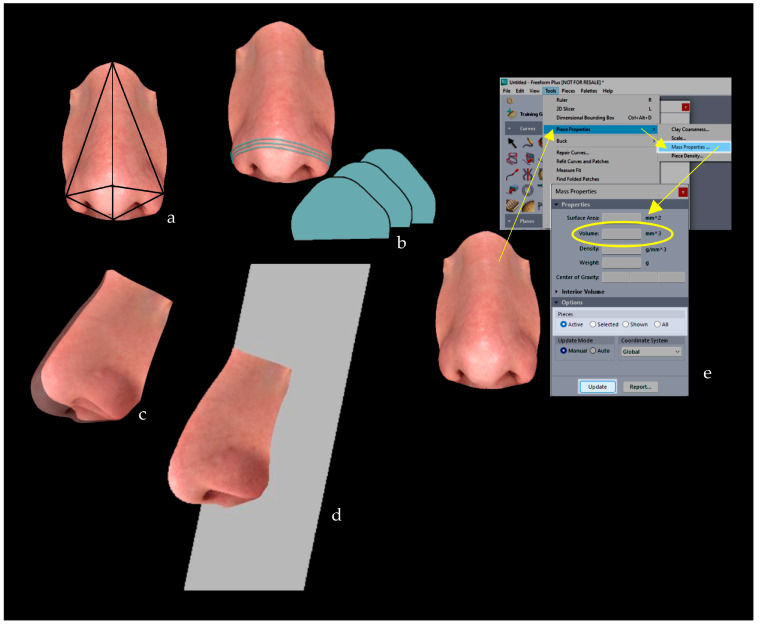
Methodologies for volume calculation. (**a**) Tetrahedra approximation; (**b**) sum of the areas over horizontal planes; (**c**) pre- (transparent nose mesh) and post- (full colour nasal mesh) superimpo-sition and registration; (**d**) superimposition and registration over an imported plane; (**e**) “piece property” function of GeoMagic.

Studies involving the volumetric calculation of maxillofacial structures can be broadly categorised into using either custom algorithms or built-in functions of the software (Figure 5 and Table 5). However, three studies [44,49,50] did not report sufficient details and therefore could not be classified. Remarkably, only 6 out of 17 studies (35%) [27,35,36,37,38,45] reported whether the protocol was validated in the study [45] or previously [27,35,36,37,38]. Additionally, six different studies [40,42,44,47,49,50] reported whether the intra- and/or inter-operator reliability was assessed.

#### 3.5.1. Custom Algorithms

Four studies developed and used their own custom algorithm to calculate the volume. Of these, three studies [35,36,37] calculated the volume of the previously selected polyhedra (Figure 5a), while one [43] summed the areas under the curve of the left and right sides of the analysed structure (nose) over horizontal planes (Figure 5b). Sforza et al. [36] calculated the volume of the whole face and its parts (forehead, maxilla, and mandible) by additionally closing the selected polyhedral structures with posterior planes (combining the methods shown in Figure 5a,d).

#### 3.5.2. Automatic Software Calculation

In most cases [27,38,39,40,41,42,45,46,47,48], the calculation of the volume of the ROI was automatically performed using the built-in functions of the software used. To calculate the volume correctly, the structure of interest must be closed three-dimensionally. Most studies used one or more imported virtual planes [27,38,40,45,47] (Figure 5d) or another reference 3D model [41,42,46,48] (Figure 5c), while only one study [39] used a custom function of the GeoMagic FreeForm Plus software (version V2017) that allows for the automatic calculation of the volume (Figure 5e).

### 3.6. Volumetric Data Reporting

A total of 13 out of 17 studies (76.5%) reported the effective volumes [27,38,39,40,44,45,47,49,50] or volume changes [41,42,46,48] in physical units (as cubic millimetres, cubic centimetres, or millilitres). In contrast, other studies [35,36,37,43] used dimensionless indices. In addition, regardless of the type of data reported, the results also differed in the type of the reported values. Most studies presented the results as the mean ± SD [35,36,37,38,40,44,45,48]. Three studies [42,46,50] were case reports without any mean values; two studies [39,47] reported volumes for each individual patient and related descriptive statistics; one study [27] described the results in terms of median and interquartile amplitude; and another reported only the mean results [43].

#### 3.6.1. Studies Reporting Effective Volumes

Nine studies [27,38,39,40,44,45,47,49,50] reported the effective volume of the structures analysed, where “effective” means the actual/real volume of the structure (Table 6). The effective value of the volume was reported for different structures, such as the nose, the palate and its segments, the maxilla, the lips, and different paranasal and perioral surfaces. However, the effective volumes reported by different studies for the same structure are not comparable because the structures belong to patients with different characteristics.

#### 3.6.2. Studies Reporting Volumetric Changes

Four studies [41,42,46,48] reported the volumetric change (Δ) of the analysed structures at two different time points, such as post- and pre-surgery (Table 7). In these studies, the analysed structures consisted of the whole face or the middle and lower thirds of the face.

#### 3.6.3. Studies Reporting Dimensionless Indices

The remaining four studies [35,36,37,43] used dimensionless indices to express the volumetric discrepancies between dysmorphic and normal healthy maxillofacial structures (Table 8). Specifically, three studies [35,36,37] used Z-scores and another [43] used a custom index defined as “Tip-Alar volume ratio”. The dimensionless indices helped to characterize several facial structures, such as the face and facial thirds, the nose, and the lips.

## 4. Discussion

Three-dimensional optical surface imaging is a technique widely used for maxillofacial analysis in the fields of morphology, dysmorphology, dentistry, and surgery. Non-radiation methods such as 3D-surface scanning provide highly accurate images of superficial facial structures and are preferable to invasive methods when the diagnostic and clinical purposes concern facial soft tissues and their changes, such as after surgery or during growth [51]. The 3D optical surface methods provide accurate 3D images that can be evaluated both qualitatively and quantitatively by performing a variety of clinically relevant measurements, including volumetric assessments. Accurate volumetric measurements of the maxillofacial regions may be required for various purposes, such as preoperative planning in both reconstructive and aesthetic procedures or the characterisation of dysmorphic facial phenotypes of syndromic patients [52,53,54,55]. The 3D surface imaging techniques have overcome the limitations of 2D techniques and have revolutionised the field with their safety, ease of use, reliability, high speed, and improved portability [56]. However, 3D analysis of facial areas and structures remains underused due to the high cost of the instruments and the need for specialized operators able to use the devices and manipulate the 3D images [57].

Although the importance of 3D volumetric analyses of dysmorphic maxillofacial regions in the clinical setting has been recently highlighted [58,59,60], 3D volumetric analyses are still rarely employed because of the lack of high-level evidence in the literature demonstrating its reliability, repeatability, and utility in practice. There is currently no reliable gold standard for the objective measurement of the volume of complex 3D maxillofacial structures captured by 3D surface imaging techniques [61]. This scoping review aimed to map the existing literature on the 3D volumetric analysis of maxillofacial structures in dysmorphic patients and to provide, whenever possible, reference data. This scoping review included 17 articles published annually between 2004 and 2023, revealing a constant research interest on this topic over the time. Its findings highlighted that the volumetric data of dysmorphic maxillofacial structures available in the literature are greatly heterogeneous.

Most studies analysed patients with CL/P [27,38,39,43,44,45,47,48,49,50], which represented the majority of the study sample of this review (69% of 476 patients), while fewer considered a specific syndrome [35,36,37,40,41,42,46] except for Down Syndrome, which received more attention, but from the same research group [35,37]. In contrast, studies focusing on syndromic patients analysed only one [41,42,46] or a maximum of five subjects [41]. The greater interest in CL/P patients can be explained by the need to surgically treat those anatomical structures that are fundamental to essential functionalities, such as feeding, breathing, swallowing, and phonation [62,63], as also evidenced by the typology of these studies, which were always surgical save for one [44]. Nonetheless, although aiming for similar purposes, the volume calculation methods and the protocol used to select the 3D anatomical structures implemented by these studies were diverse, indicating that no preferred methods exist in the case of surgical studies focusing on CL/P patients. Conversely, the studies analysing syndromic patients were balanced in terms of purposes (whether “surgical” or “morphological”): the 3D volumetric analysis, again, was quite heterogenous except for the surgical studies [41,42,46] that evaluated volumetric changes of the entire face or its parts after a surgical intervention, although these used different protocols of selection. These findings indicate a great heterogeneity between the studies in terms of the patients (e.g., age and pathology) and the maxillofacial structures analysed, and thus, there are numerous protocols used to select the ROI and to calculate its volume. Despite the heterogeneity, once the maxillofacial structure has been three-dimensionally acquired, the volumetric calculation always involves several prior steps, and the one that seems to impact the final volumetric data most is the ROI selection protocol. However, the lack of comparable data for the same anatomical structures, selected with different protocols, prevents any possibility to verify this aspect, and this assertion remains a speculation to be confirmed or excluded in future studies. Overall, the included studies also differed in terms of the technology and systems used for the 3D scansion and reproduction of the anatomical structures. Different instruments have different characteristics in terms of precision, accuracy, and repeatability, which may have a non-negligible influence on the final volumetric data. In other words, comparative studies assessing the interchangeability of devices in reproducing the same 3D anatomical structure should be performed to verify whether the volumetric data are equivalent. Although this goal seems easily achievable, one must consider the constant and growing release of new instruments and novel technologies on the market [64].

Another important finding highlighted by this review is the number and variety of protocols used for calculating the volume, regardless of the type of structures analysed and the instrument used for its acquisition, but with the latter often leading to the choice of which software to use for the analysis. In fact, 47% of the studies used the software developed by the manufacturer of the scanning instrument used, and additional software were only sometimes used to complete the volume calculation protocol. Again, we did not find a preferred method of volume calculation in terms of the instrument or software used, and in fact, we have uncovered different methods using either volume approximation by polyhedron or contours/curvatures analysis [35,36,37,43] or automatic volume calculation by a software. In particular, the software can calculate effective volumes when the ROI is registered on a virtual plane imported to close it [27,38,40,45,47] or volumetric changes when the ROI is registered on another relevant ROI for comparison [41,42,46,48], such as in pre- and post-surgery 3D image superimpositions. Only one study [39] used a specific function of the GeoMagic Freeform Plus software to automatically determine the volume, avoiding any registration onto a plane or a to a different ROI. It is also worth noting that three studies [44,49,50] did not report enough details on the actual volumetric calculation, hindering the possibility to determine whether other protocols are available. Furthermore, the volumetric calculation protocol used in the analysis seems to be independent of the used software, as different protocols can be performed by each software, leaving the choice of the protocol to the researchers.

To summarize the findings of this scoping review presented so far, the scanning technology, the instrument, the software, and the 3D volumetric analysis (including the selection of the ROI and the calculation of its volume), in addition to the study characteristics already mentioned, all contributed to the heterogeneity observed in all of the included studies.

Although a direct comparison between the reported data was not possible, and therefore, the interchangeability between different methodologies could not be verified, these data could potentially be useful for researchers and clinicians working in this field, serving as ‘reference’ or comparative data. Yet, caution is still needed in their use, as few studies reported volume measurements calculated with validated protocols [27,35,36,37,38,45]. Future studies are needed to verify eventual differences due to the protocol for the ROI selection and the automatic volume calculation by different software and protocols.

This scoping review is not without limitations, the most important of which was the impossibility of verifying the equivalence of the data reported by the included studies. Another limitation is the inclusion of studies using only 3D optical methods, yet the research question was specific on this aspect, as these technologies are becoming the gold standard for the analysis of the superficial soft tissues [18], increasingly replacing the use of radiodiagnostic methods such as MRI and CT. Indeed, we are aware that further information and results could have been obtained by including studies using radiological imaging techniques. Finally, the review focused on the maxillofacial structures of dysmorphic or syndromic patients. Data on either other anatomical structures or healthy subjects still need to be reviewed.

## 5. Conclusions

This scoping review has highlighted the existence of several 3D volumetric protocols that can be applied to the anthropometric analysis of dysmorphic maxillofacial structures. Therefore, different methods may provide inconsistent and non-interchangeable volumetric data, which highlights the need to investigate this important issue for its clinical relevance. The volumetric data in the literature could theoretically be used as reference; however, this is not yet recommended in practice because of the lack of a methodological consensus and of sound scientific evidence on the comparability and interchangeability of methods and data. Thus, the message our scoping review aims to convey to the research community is to unite efforts to create homogeneity in the proposed protocols and data provided in the literature in order to tune reliable 3D volumetric analyses for the clinical evaluation of maxillofacial structures. In our opinion, the 3D representation and objective quantification of the volume of dysmorphological maxillofacial structures represent important future goals to enhance morphological understanding, clinical practice, and surgical and diagnostic applications.

## Figures and Tables

**Table 1:** Search strategy.

Scopus	
TITLE-ABS-KEY(“3D imaging” OR “Laser scan*” OR “Facial imaging” OR “Stereophotogrammetry” OR “Surface imaging” OR “Topograph*”) AND ALL (volumetr* OR “volume measur*” OR soft tissue measurement) AND ALL(“Cleft lip” OR “Cleft palate” OR syndrom* OR dysmorph* OR disorder* OR deform*) AND PUBYEAR AFT 2002	
Embase	
‘3d imag*’ OR ‘3-d imag*’ OR ‘three-dimensional imag*’ OR ‘laser scan*’ OR ‘facial imaging’ OR ‘stereophotogrammetry’/exp OR ‘stereophotogrammetry’ OR ‘surface imaging’ OR ‘topograph*’	#1
‘volumetr*’ OR ‘volume measur*’ OR ‘soft tissue measurement’	#2
‘cleft lip’ OR ‘cleft palate’ OR ‘syndrom’ OR ‘dysmorph*’ OR ‘disorder*’ OR ‘deform*’	#3
#1 AND #2 AND #3 AND [2002–2024]/py	#4
PubMed	
((“3D imaging” OR “laser scan*” OR “Facial imaging” OR “Stereophotogrammetry” OR “imaging, three dimensional” OR “Surface imaging” OR “topograph*”) AND (“volumetr*” OR “volume measur*” OR (“soft” AND (“tissue s” OR “tissues” OR “tissues” OR “tissue”) AND (“measurability” OR “measurable” OR “measurably” OR “measure s” OR “measureable” OR “measured” OR “measurement” OR “measurement s” OR “measurements” OR “measurer” OR “measurers” OR “measuring” OR “measurings” OR “measurment” OR “measurments” OR “weights and measures” OR (“weights” AND “measures”) OR “weights and measures” OR “measure” OR “measures”))) AND (“Cleft lip” OR “Cleft palate” OR “syndrom*” OR “dysmorph*” OR “disorder*” OR “deform*”)) AND (2002:2024[pdat])	
Web of Science	
ALL = (“3D imag*” OR “3-D imag*” OR “Three-dimensional imag*” OR “Laser scan*” OR “Facial imaging” OR “Stereophotogrammetry” OR “Surface imaging” OR “Topograph*”)	#1
ALL = (volumetr* OR “volume measur*” OR soft tissue measurement)	#2
ALL = (“Cleft lip” OR “Cleft palate” OR syndrom* OR dysmorph* OR disorder* OR deform*)	#3
#1 AND #2 AND #3 and 2002 or 2003 or 2004 or 2005 or 2006 or 2007 or 2008 or 2009 or 2010 or 2011 or 2012 or 2013 or 2014 or 2015 or 2016 or 2017 or 2018 or 2019 or 2020 or 2021 or 2022 or 2023 or 2024 (Publication Years)	#4

**Table 2:** Number of studies and patients for each age class considered.

Patients Age-Class		Number of Studies	Number of Patients
Only Children (<13 yo)	I + P + S	7	269
Only Teenagers (13 to 18 yo)	T	2	2
Only Adults (≥18 yo)	A	1	1
Subjects < 18 yo	C + T	1	12 (10C, 2T)
Subjects ≥ 13 yo	T + A	2	23
Subadults and adults	C + T + A	4	169
Total		17	476

I: infants (0–2 years old); P: preschool children (3–6 years old); S: school-aged children (6–12 years old); C: Children (<13 years old: I + P + S), T: teenagers (13–18 years old); A: adults (18+ years old); yo: years old.

**Table 3:** Number of studies and patients for each analysed pathology.

Pathology		Number of Studies	Number of Patients
Cleft Lip and/or Palate	Unilateral Cleft Lip	10	143
	Unilateral Cleft Lip and Palate		108
	Bilateral Cleft Lip		14
	Bilateral Cleft Lip and Palate		63
Total CL/P		10	328
Syndromes	Down	2	92
	Moebius	1	26
	Hemifacial microsomia	1	1
	Parry-Romberg	1	1
	Marfan	1	16
	Craniosynostosis: Crouzon Apert Pfeiffer Saethre–Chotzen Unknown	1	12: 5 3 2 1 1
Total Syndromes		7	148
Total		17	476

**Table 4:** Demographic information of the patients included in the studies.

First Author (Year of Publication)	Pathology/ Syndrome	Sample Size	Age Group of Patients	Mean Age	Number of Females (% of Females)	Ethnicity/ Country of Origin
Ferrario et al. (2004) [35]	Down syndrome	28	S, T, A	Males: 26.4 ± 9.4 yo Range: 12–41 yoFemales: 27.5 ± 8.9 yo Range: 15–45 yo	11 (39%)	Northern Italy
Sforza et al. (2009) [36]	Moebius syndrome	26	P, S, T, A	17 ± 14 yo Range: 3–52 yo	14 (54%)	NA
Jayaratne et al. (2010) [42]	Hemifacial microsomia	1	A	19 yo	1 (100%)	Asian *
Van Loon et al. (2010) [49]	UCL and UCLP	Total 12	T, A	18 ^§^ yo Range: 13–40 yo	4 (33%)	NA
		UCL 2				
		UCLP 10				
Sforza et al. (2011) [37]	Down syndrome	64	P, S, T, A	15 ± 7 yo Range 4–34 yo	18 (28%)	North Sudan with North African origins
Chan et al. (2013) [41]	Crouzon, Apert, Pfeiffer, Saethre-Chotzen syndromes	12	S, T	10.1 yo	NA	NA
Pucciarelli et al. (2015) [45]	UCLP	32	I	10.5 ± 4.8 days	15 (47%)	NA
Susarla et al. (2015) [48]	UCLP and BCLP	Total 11	T, A	17.9 ± 1.3 yo	4 (36%)	NA
		UCLP 6				
		BCLP 5				
Vaughan et al. (2016) [50]	UCLP	1	T	14 yo	NA	NA
Mercan et al. (2018) [43]	UCL	Total 89	I, S		30 (34%)	Mixed: Caucasian 44 Asian 23 Native American 1 Mixed Caucasian 7 Other 9 Not specififed 5
		Group A 45		Group A Pre-surgery (T1) 7.5 mo Post-surgery (T2) 10 mo	Group A 13 (29%)	
		Group B 44		Group B Post-surgery 9.5 yo	Group B 17 (39%)	
Ozdemir et al. (2018) [44]	UCLP and BCLP	Total 51	S, T, A		NA	NA
		UCLP 29		UCLP group: 15.45 ± 5.15 yo		
		BCLP 22		BCLP group: 16.18 ± 5.89 yo		
Paoloni et al. (2018) [40]	Marfan syndrome	16	S	8.8 ± 1.5 yo	7 (49%)	Caucasian
Pucciarelli et al. (2018) [46]	Parry-Romberg syndrome	1	T	15 yo	0 (0%)	Caucasian *
Rizzo et al. (2019) [47]	UCLP	10	I	3 mo	3 (30%)	NA
Ambrosio et al. (2021) [38]	BCL and BCLP	Total 50	I	Pre-surgery (T1): 0.41 ± 0.16 yo	NA	NA
		BCL 14		Post-cheiloplasty (T2): 1.33 ± 0.33 yo		
		BCLP 36		Post-palatoplasty (T3): 2.45 ± 0.45 yo		
Ambrosio et al. (2022) [27]	UCL and UCLP	Total 41	I	Pre-surgery (T1): 0.35 ± 0.07 yo	17 (41%)	NA
		UCL 21		Post-cheiloplasty (T2): 1.30 ± 0.18 yo	UCL 11 (52%)	
		UCLP 20		Post-palatoplasty (T3): 2.1 ± 0.22 yo	UCLP 6 (30%)	
Chattopadhyay et al. (2023) [39]	UCL	31	I	Pre-surgery (T1): 5 mo range: 3–8 mo Post-surgery (T2): after 3 weeks Post-surgery (T3): after 2 years	13 (42%)	NA

I: infants (0–2 years old); P: preschool children (3–6 years old); S: school-aged children (6–12 years old), T: teenagers (13–18 years old); A: adults (18+ years old). ^§^: median; yo: years old; mo: months old. UCL: unilateral cleft-lip; UCLP: unilateral cleft-lip and palate; BCLP: bilateral cleft-lip and palate; CLP: cleft lip and palate. NA: information not available; *: Ethnicity not specified in the case report article but deducible from the patient’s pictures.

**Table 5:** 3D technology, instrument, software, and protocol used for the volumetric analysis.

First author (Year of Publication)	Pathology/ Syndrome	Scanning System Type	Type of Acquisition	Software Associate with Device	Software	Structures Analysed	ROI Selection Protocol	Volume Calculation Protocol	Protocol Validation
Ferrario et al. (2004) [35]	Down syndrome	Electromechanical digitizer (Microscribe G2)	Direct acquisition	No	In-house-built software	Lip and Nose	Use of anatomical landmarks	Approximation with polyhedra using a custom computer program for offline calculation	Yes, in previous studies
Sforza et al. (2009) [36]	Moebius syndrome	Electromechanical digitizer (Microscribe G2)	Direct acquisition	No	In-house-built software	Whole face, forehead, maxilla, mandible and nose	Use of anatomical landmarks and planes	Approximation with polyhedra using a custom computer program for offline calculation	Yes, in previous studies
Jayaratne et al. (2010) [42]	Hemifacial Microsomia	Stereophotogrammetry (3dMD face)	Direct acquisition	Yes	Manufacturer software	Maxilla and mandibular areas	NA	Registration and superimposition of post- on pre-operative facial image to automatically calculate volumetric changes	Only reliability
Van Loon et al. (2010) [49]	UCL and UCLP	Stereophotogrammetry (3dMD face)	Direct acquisition	Yes, partially	Manufacturer software (3dMDpatient version 3.0.1) and Maxilim software version 2.2.2.1	Nose	Use of landmarks-based planes	NA	Only reliability
Sforza et al. (2011) [37]	Down syndrome	Laser scanning (FastSCAN Cobra)	Direct acquisition	No	Rhinoceros Nurbs for Windows 4.0 software	Nasolabial area	Use of anatomical landmarks	Approximation with polyhedra using a custom computer program for offline calculation	Yes, in previous studies
Chan et al. (2013) [41]	Crouzon, Apert, Pfeiffer, Saethre–Chotzen syndromes	Stereophotogrammetry (MU-4 Imaging System)	Direct acquisition	Yes	Manufacturer software	Whole face	Entire 3D model	Registration and superimposition of post- on pre-operative facial image to automatically calculate volumetric changes	Unspecified
Pucciarelli et al. (2015) [45]	UCLP	Stereophotogrammetry (VECTRA 3D)	Indirect acquisition from casts	Yes	Manufacturer software	Palate	Use of contouring points	Projection of the ROI’s points on a virtual plane, registration of the two surfaces for closing the ROI, and automatic calculation of the volume	Yes
Susarla et al. (2015) [48]	CLP	Stereophotogrammetry (Vectra XT)	Direct acquisition	Yes	Manufacturer software	Nasolabial area	Use of anatomical landmarks and manual selection	Registration and superimposition of post- on pre-operative facial image to automatically calculate volumetric changes	Unspecified
Vaughan et al. (2016) [50]	UCLP	Stereophotogrammetry (3dMD face)	Direct acquisition	No	Rapidform 2006 and Invivo version 5.2.3 software	Maxilla	NA	NA	Only reliability
Mercan et al. (2018) [43]	UCL	Stereophotogrammetry (3dMD cranial system)	Direct acquisition	No	In-house-built software	Nose	Use of horizontal contours and reference planes	Approximation of volume as the sum of the areas under left and right sides over all reference planes (own calculation through contours/curvature analysis)	Unspecified
Ozdemir et al. (2018) [44]	UCLP and BCLP	Stereophotogrammetry (3dMD face)	Direct acquisition	Yes	Manufacturer software (3dMDvultus version 2.3.0.2)	Nasolabial area and chin	Use of horizontal and vertical reference planes	NA	Only reliability
Paoloni et al. (2018) [40]	Marfan syndrome	Laser scanning (OrthoXscan)	Indirect acquisition from casts	NA	NA	Palate	Use of landmarks-based planes	Virtual volume enclosed by the digital casts and planes used for ROI selection	Only reliability
Pucciarelli et al. (2018) [46]	Parry–Romberg Syndrome	Stereophotogrammetry (VECTRA 3D)	Direct acquisition	Yes	Manufacturer software	Trigeminal facial thirds	Use of anatomical landmarks	Registration and superimposition of post- on pre-operative facial image to automatically calculate volumetric changes	Unspecified
Rizzo et al. (2019) [47]	UCLP	Stereophotogrammetry (3dMD trio)	Direct acquisition	Yes, partially	Manufacturer software and GeoMagic Wrap	Upper lip	Use of anatomical landmarks	Use of a virtual plane for closing the ROI, and automatic computation of the volume	Only reliability
Ambrosio et al. (2021) [38]	CLP	Laser scanning (R700 Scanner)	Indirect acquisition from casts	No	Mirror Imaging Software	Palate	Use of contouring points	Projection of the ROI’s points on a virtual plane, registration of the two surfaces for closing the ROI, and automatic calculation of the volume [45]	Yes, in previous studies
Ambrosio et al. (2022) [27]	UCL and UCLP	Laser scanning (R700 Scanner)	Indirect acquisition from casts	No	Mirror Imaging Software	Palate	Use of contouring points	Projection of the ROI’s points on a virtual plane, registration of the two surfaces for closing the ROI, and automatic calculation of the volume [45]	Yes, in previous studies
Chattopadhyay et al. (2023) [39]	UCL	Laser Scanning (Artec Space Spider)	Direct acquisition	No	GeoMagic Freeform Plus software version V2017	Nose	Use of anatomical landmarks-based planes	Automatic calculation of the ROI’s volume using the “Piece Property” function of the software	Unspecified

NA: Not applicable (i.e., no details reported).

**Table 6:** Volumetric measurements of the studies which reported the effective volumes of the analysed structures.

Author	Pathology/ Syndrome	N	Structure Analysed	Volume Pre-Surgery (cm^3^)	Volume Post-Surgery (cm^3^)
Van Loon et al. (2010) [49]	UCL	12	Cleft side nose	T1: 16.49 (3.87) ^#^	T2: 17.45 (4.31) ^#^
	UCLP		Non-cleft side nose	T1: 18.59 (4.79) ^#^	T2: 18.86 (4.73) ^#^
Pucciarelli et al. (2015) [45]	UCLP	16	Greater palatal segment after PNAM	T1: 1.08 (0.47)	T2: 1.09 (0.49) T3: 1.22 (0.56)
			Minor palatal segment after PNAM	T1: 0.53 (0.25)	T2: 0.48 (0.26) T3: 0.65 (0.29)
		16	Greater palatal segment after Hotz’s	T1: 0.91 (0.29)	T2: 1.09 (0.37) T3: 1.38 (0.51)
			Minor palatal segment after Hotz’s	T1: 0.52 (0.21)	T2: 0.68 (0.23) T3: 0.81 (0.25)
Vaughan et al. (2016) [50]	UCLP	1	Maxilla	T1: 0.36	T2: 0.41
Ozdemir et al. (2018) [44]	UCLP	29	Upper lip	NA	T2: 2.43 (1.03)
			Lower lip	NA	T1: 3.08 (1.28)
			Nose	NA	T2: 12.44 (3.81)
			Upper lip and paranasal area	NA	T2: 27.7 (5.83)
			Upper lip and paranasal area without nose	NA	T2: 15.19 (4.01)
			Lower lip and chin	NA	T2: 4.55 (2.46)
	BCLP	22	Upper lip	NA	T2: 2.52 (1.11)
			Lower lip	NA	T2: 3.44 (1.21)
			Nose	NA	T2: 13.31 (4.09)
			Upper lip and paranasal area	NA	T2: 28.44 (4.28)
			Upper lip and paranasal area without nose	NA	T2: 16.12 (3.62)
			Lower lip and chin	NA	T2: 5.93 (3.16)
Paoloni et al. (2018) [40]	Marfan syndrome	16	Palate	T1: 2.58 (0.59)	NA
Rizzo et al. (2019) [47]	UCL	10	Upper lip	T1: 1.64^§^	T2: 2.12^§^
Ambrosio et al. (2021) [38]	BCL	14	Sum of palatal segments and palatal arch	T1: 0.97 (0.77)	T2: 2.07 (0.77)
	BCLP	36	Sum of palatal segments	T1: 3.22 (0.91)	T2: 4.40 (1.26) T3: 2.68 (0.97)
Ambrosio et al. (2022) [27]	UCL	21	Sum of palatal segments	T1: 0.58 (0.76) *	T2: 1.48 (2.72) *
	UCLP	20	Sum of palatal segments	T1: 1.65 (0.99) *	T2: 3.05 (1.33) * T3: 2.25 (1.43) *
Chattopadhyay et al. (2023) [39]	UCL	31	Cleft side nose	T1: 0.004 ^§^	T2: 0.005 ^§^ T3: 0.03 (0.002)
		31	Non-cleft side nose	NA	T3: 0.03 (0.002)

Data are reported as the mean (SD) unless differently reported: ^#^ mean (SD) not provided by the original article and calculated from the authors; ^§^ mean only; * median (interquartile amplitude, IA). Post-surgery times are different time intervals for each study.

**Table 7:** Volumetric measurements of the studies reporting volumetric changes superimposing post- and pre-surgery 3D images.

Author	Pathology/ Syndrome	N	Structure Analysed	Volumetric Changes between Post- and Pre-Surgery 3D Model (cm^3^)
Jayaratne et al. (2010) [42]	Hemifacial Microsomia	1	Mid-lower face (left side)	T1_Mirrored_-T1: −16.41 T2-T1: 20.23 T3-T1: 30.84 T4-T1: 27.08 T5-T1: 23.81 T6-T1: 22.38 T7-T1: 21.43
Chan et al. (2013) [41]	Crouzon syndrome	5	Face	T2-T1: 107 (14) ^#^ T3-T1: 102 (12) ^#^
	Apert syndrome	3		T2-T1: 92 (5.5) ^#^ T3-T1: 88 (5.5) ^#^
	Pfeiffer syndrome	2		T2-T1: 101.5 (11) ^#^ T3-T1: 95.5 (8) ^#^
	Saethre–Chotzen syndrome	1		T2-T1: 74 T3-T1: 71
	Unknown syndrome	1		T2-T1: 105 T3-T1: 102
Susarla et al. (2015) [48]	UCLP	11	Midface	T2-T1: 12.2 (5.7)
	BCLP			
Pucciarelli et al. (2018) [46]	Parry–Romberg syndrome	1	Middle third (trigeminal)	T2-T1: 1.6
			Lower third (trigeminal)	T2-T1: 2.5
			Middle + Lower third	T2-T1: 4.1

Data are reported as the mean (SD) unless differently reported: ^#^ mean (SD) not provided by the original article and calculated from the authors. Post-surgery times are different time-intervals for each study.

**Table 8:** Dimensionless indices for the volumetric comparison of the structure of interest with the contralateral control side or with control subjects.

Author	Pathology/ Syndrome	N	Structure Analysed	Comparison with Control Side or Subjects (Dimensionless Index)
Ferrario et al. (2004) [35]	Down Syndrome	28	Nose	−1.31 (1.19) ^Z^
			Upper lip	0.07 (1.12) ^Z^
			Lower lip	1.04 (0.71) ^Z^
			Lips	−0.51 (0.68) ^Z^
Sforza et al. (2009) [36]	Moebius Syndrome	26	Face	−0.38 (1.14) ^Z^
			Forehead	−0.45 (1.19) ^Z^
			Maxilla	0.17 (1.03) ^Z^
			Mandible	−0.78 (1.27) ^Z^
			Nose	−0.92 (1.40) ^Z^
Sforza et al. (2011) [37]	Down Syndrome	64	Nose	−0.20 (1.12) ^Z^
			Upper Lip	−0.09 (1.33) ^Z^
			Lips	−0.40 (1.31) ^Z^
Mercan et al. (2018) [43]	CL	45	Nose	T1: 1.59 ^§R^ T2: 1.20 ^§R^
		44		T2: 1.18 ^§R^

Data are reported as the mean (SD) unless differently reported: ^§^ mean only. The indices used are ^Z^ Z-scores and ^R^ Tip–Alar volume ratio. Post-surgery times are different time intervals for each study.

## Data Availability

The data presented in this study are available on request from the corresponding author.

## References

[1] Figueroa D.A.A., Friede D.H. (2000). Craniofacial Growth in Unoperated Craniofacial Malformations. Cleft Palate-Craniofacial J..

[2] Hunt J.A., Hobar P.C. (2003). Common Craniofacial Anomalies: Conditions of Craniofacial Atrophy/Hypoplasia and Neoplasia. Plast. Reconstr. Surg..

[3] Cronin A., McLeod S., Damico J.S., Ball M.J. (2019). Craniofacial Anomalies. The SAGE Encyclopedia of Human Communication Sciences and Disorders.

[4] Hunter A.G.W. (2002). Medical Genetics: 2. The Diagnostic Approach to the Child with Dysmorphic Signs. CMAJ.

[5] Van Den Elzen M.E.P., Versnel S.L., Hovius S.E.R., Passchier J., Duivenvoorden H.J., Mathijssen I.M.J. (2012). Adults with Congenital or Acquired Facial Disfigurement: Impact of Appearance on Social Functioning. J. Cranio-Maxillofac. Surg..

[6] Hickey A.J., Salter M. (2006). Prosthodontic and Psychological Factors in Treating Patients with Congenital and Craniofacial Defects. J. Prosthet. Dent..

[7] Wiechers C., Thjen T., Koos B., Reinert S., Poets C.F. (2021). Treatment of Infants with Craniofacial Malformations. Arch. Dis. Child. Fetal Neonatal Ed..

[8] American Cleft Palate–Craniofacial Association (2018). Parameters: For Evaluation and Treatment of Patients with Cleft Lip/Palate or Other Craniofacial Differences. Cleft Palate-Craniofacial J..

[9] Bohm L.A., Sidman J.D., Roby B. (2016). Early Airway Intervention for Craniofacial Anomalies. Facial Plast. Surg. Clin. N. Am..

[10] Prahl-Andersen B. (2005). Controversies in the Management of Craniofacial Malformations. Semin. Orthod..

[11] Trainor P.A., Richtsmeier J.T. (2015). Facing up to the Challenges of Advancing Craniofacial Research. Am. J. Med. Genet. A.

[12] Mai H.N., Kim J., Choi Y.H., Lee D.H. (2021). Accuracy of Portable Face-Scanning Devices for Obtaining Three-Dimensional Face Models: A Systematic Review and Meta-Analysis. Int. J. Environ. Res. Public. Health.

[13] Cen Y., Huang X., Liu J., Qin Y., Wu X., Ye S., Du S., Liao W. (2023). Application of Three-Dimensional Reconstruction Technology in Dentistry: A Narrative Review. BMC Oral. Health.

[14] Karatas O.H., Toy E. (2014). Three-Dimensional Imaging Techniques: A Literature Review. Eur. J. Dent..

[15] Heike C.L., Upson K., Stuhaug E., Weinberg S.M. (2010). 3D Digital Stereophotogrammetry: A Practical Guide to Facial Image Acquisition. Head. Face Med..

[16] Honrado C.P., Larrabee W.F. (2004). Update in Three-Dimensional Imaging in Facial Plastic Surgery. Curr. Opin. Otolaryngol. Head. Neck Surg..

[17] Lo L.J., Lin H.H. (2023). Applications of Three-Dimensional Imaging Techniques in Craniomaxillofacial Surgery: A Literature Review. Biomed. J..

[18] D’Ettorre G., Farronato M., Candida E., Quinzi V., Grippaudo C. (2022). A Comparison between Stereophotogrammetry and Smartphone Structured Light Technology for Three-Dimensional Face Scanning. Angle Orthod..

[19] Gibelli D., Dolci C., Cappella A., Sforza C. (2020). Reliability of Optical Devices for Three-Dimensional Facial Anatomy Description: A Systematic Review and Meta-Analysis. Int. J. Oral. Maxillofac. Surg..

[20] Gibelli D., Cappella A., Dolci C., Sforza C. (2019). 3D Surface Acquisition Systems and Their Applications to Facial Anatomy: Let’s Make a Point. Ital. J. Anat. Embryol..

[21] Kau C., Zhurov A., Scheer R., Bouwman S., Richmond S. (2004). The Feasibility of Measuring Three-dimensional Facial Morphology in Children. Orthod. Craniofac Res..

[22] Primozic J., Ovsenik M., Richmond S., Kau C.H., Zhurov A. (2009). Early Crossbite Correction: A Three-Dimensional Evaluation. Eur. J. Orthod..

[23] Gibelli D., Tarabbia F., Restelli S., Allevi F., Dolci C., Dell’Aversana Orabona G., Cappella A., Codari M., Sforza C., Biglioli F. (2020). Three-Dimensional Assessment of Restored Smiling Mobility after Reanimation of Unilateral Facial Palsy by Triple Innervation Technique. Int. J. Oral. Maxillofac. Surg..

[24] Da Pozzo F., Gibelli D., Beltramini G.A., Dolci C., Giannì A.B., Sforza C. (2020). The Effect of Orthognathic Surgery on Soft-Tissue Facial Asymmetry: A Longitudinal Three-Dimensional Analysis. J. Craniofacial Surg..

[25] Farkas L.G., Deutsch C.K. (1996). Anthropometric Determination of Craniofacial Morphology. Am. J. Med. Genet..

[26] Farkas L.G. (1994). Anthropometry of the Head and Face.

[27] Ambrosio E.C.P., Fusco N.D.S., Carrara C.F.C., Bergamo M.T., Lourenço Neto N., Cruvinel T., Rios D., Almeida A.L.P.F., Soares S., Machado M.A.A.M. (2022). Digital Volumetric Monitoring of Palate Growth in Children with Cleft Lip and Palate. J. Craniofacial Surg..

[28] Hohoff A., Stamm T., Meyer U., Wiechmann D., Ehmer U. (2006). Objective Growth Monitoring of the Maxilla in Full Term Infants. Arch. Oral. Biol..

[29] Ackerman J.L., Proffit W.R., Sarver D.M. (1999). The Emerging Soft Tissue Paradigm in Orthodontic Diagnosis and Treatment Planning. Clin. Orthod. Res..

[30] Proffit W.R. (2000). The Soft Tissue Paradigm in Orthodontic Diagnosis and Treatment Planning: A New View for a New Century. J. Esthet. Dent..

[31] Jansma J., Schepers R.H. (2023). Adjunctive Aesthetic Procedures in Orthognathic Surgery. Oral. Maxillofac. Surg. Clin. N. Am..

[32] Tricco A.C., Lillie E., Zarin W., O’Brien K.K., Colquhoun H., Levac D., Moher D., Peters M.D.J., Horsley T., Weeks L. (2018). PRISMA Extension for Scoping Reviews (PRISMA-ScR): Checklist and Explanation. Ann. Intern. Med..

[33] Ouzzani M., Hammady H., Fedorowicz Z., Elmagarmid A. (2016). Rayyan—A Web and Mobile App for Systematic Reviews. Syst. Rev..

[34] Peters M.D.J., Marnie C., Tricco A.C., Pollock D., Munn Z., Alexander L., McInerney P., Godfrey C.M., Khalil H. (2020). Updated Methodological Guidance for the Conduct of Scoping Reviews. JBI Evid. Synth..

[35] Ferrario V.F., Dellavia C., Colombo A., Sforza C. (2004). Three-Dimensional Assessment of Nose and Lip Morphology in Subjects with Down Syndrome. Ann. Plast. Surg..

[36] Sforza C., Grandi G., Pisoni L., Di Blasio C., Gandolfini M., Ferrario V.F. (2009). Soft Tissue Facial Morphometry in Subjects with Moebius Syndrome. Eur. J. Oral. Sci..

[37] Sforza C., Elamin F., Rosati R., Lucchini M.A., Tommasi D.G., Ferrario V.F. (2011). Three-Dimensional Assessment of Nose and Lip Morphology in North Sudanese Subjects with Down Syndrome. Angle Orthod..

[38] Ambrosio E.C.P., Sforza C., de Menezes M., Carrara C.F.C., Soares S., Machado M.A.A.M., Oliveira T.M. (2021). Prospective Cohort 3D Study of Dental Arches in Children with Bilateral Orofacial Cleft: Assessment of Volume and Superimposition. Int. J. Paediatr. Dent..

[39] Chattopadhyay D., Kapoor A., Vathulya M., Bera S. (2023). Volumetric Assessment of the Nose after Primary Unilateral Cleft Rhinoplasty Using Laberge’s Technique. J. Plast. Reconstr. Aesthet. Surg..

[40] Paoloni V., Cretella Lombardo E., Placidi F., Ruvolo G., Cozza P., Laganà G. (2018). Obstructive Sleep Apnea in Children with Marfan Syndrome: Relationships between Three-Dimensional Palatal Morphology and Apnea-Hypopnea Index. Int. J. Pediatr. Otorhinolaryngol..

[41] Chan F.C., Kawamoto H.K., Federico C., Bradley J.P. (2013). Soft-Tissue Volumetric Changes Following Monobloc Distraction Procedure. J. Craniofacial Surg..

[42] Jayaratne Y.S.N., Lo J., Zwahlen R.A., Cheung L.K. (2010). Three-dimensional Photogrammetry for Surgical Planning of Tissue Expansion in Hemifacial Microsomia. Head. Neck.

[43] Mercan E., Morrison C.S., Stuhaug E., Shapiro L.G., Tse R.W. (2018). Novel Computer Vision Analysis of Nasal Shape in Children with Unilateral Cleft Lip. J. Cranio-Maxillofac. Surg..

[44] Ozdemir A.S., Esenlik E. (2018). Three-Dimensional Soft-Tissue Evaluation in Patients with Cleft Lip and Palate. Med. Sci. Monit..

[45] Pucciarelli V., Pisoni L., De Menezes M., Ceron Zapata A.M., Lopez-Palacio A.M., Codari M., Sforza C. Palatal Volume Changes in Unilateral Cleft Lip and Palate Paediatric Patients. Proceedings of the 6th International Conference on 3D Body Scanning Technologies.

[46] Pucciarelli V., Baserga C., Codari M., Beltramini G.A., Sforza C., Giannì A.B. (2018). Three-Dimensional Stereophotogrammetric Evaluation of the Efficacy of Autologous Fat Grafting in the Treatment of Parry-Romberg Syndrome. J. Craniofacial Surg..

[47] Rizzo M.I., Zadeh R., Bucci D., Palmieri A., Monarca C., Staderini E., Oliva G., Candida E., Gallenzi P., Cordaro M. (2019). Volumetric Analysis of Cleft Lip Deformity Using 3D Stereophotogrammetry. Ann. Ital. Chir..

[48] Susarla S.M., Berli J.U., Kumar A. (2015). Midfacial Volumetric and Upper Lip Soft Tissue Changes After Le Fort I Advancement of the Cleft Maxilla. J. Oral. Maxillofac. Surg..

[49] van Loon B., Maal T.J., Plooij J.M., Ingels K.J., Borstlap W.A., Kuijpers-Jagtman A.M., Spauwen P.H., Bergé S.J. (2010). 3D Stereophotogrammetric Assessment of Pre- and Postoperative Volumetric Changes in the Cleft Lip and Palate Nose. Int. J. Oral. Maxillofac. Surg..

[50] Vaughan S.M., Kau C.H., Waite P.D. (2016). Novel Three-Dimensional Understanding of Maxillary Cleft Distraction. J. Craniofacial Surg..

[51] Heike C.L., Cunningham M.L., Hing A.V., Stuhaug E., Starr J.R. (2009). Picture Perfect? Reliability of Craniofacial Anthropometry Using Three-Dimensional Digital Stereophotogrammetry. Plast. Reconstr. Surg..

[52] Gibelli D., Pucciarelli V., Cappella A., Dolci C., Sforza C. (2018). Are Portable Stereophotogrammetric Devices Reliable in Facial Imaging? A Validation Study of VECTRA H1 Device. J. Oral. Maxillofac. Surg..

[53] Hall R.L. (2005). Energetics of Nose and Mouth Breathing, Body Size, Body Composition, and Nose Volume in Young Adult Males and Females. Am. J. Human. Biol..

[54] Tzou C.-H.J., Artner N.M., Pona I., Hold A., Placheta E., Kropatsch W.G., Frey M. (2014). Comparison of Three-Dimensional Surface-Imaging Systems. J. Plast. Reconstr. Aesthetic Surg..

[55] Fastuca R., Campobasso A., Zecca P.A., Caprioglio A. (2018). 3D Facial Soft Tissue Changes after Rapid Maxillary Expansion on Primary Teeth: A Randomized Clinical Trial. Orthod. Craniofac Res..

[56] Miranda R.E.D., Matayoshi S., Brabo J.L., Miyoshi L.H. (2018). Use of Stereophotogrammetry for Measuring the Volume of External Facial Anatomy: A Systematic Review. Rev. Bras. Cir. Plástica RBCP Braz. J. Plast. Sugery.

[57] Silva R., Silva B., Fernandes C., Morouço P., Alves N., Veloso A. (2024). A Review on 3D Scanners Studies for Producing Customized Orthoses. Sensors.

[58] Azzi A.J., Hilzenrat R., Viezel-Mathieu A., Hemmerling T., Gilardino M. (2018). A Review of Objective Measurement of Flap Volume in Reconstructive Surgery. Plast. Reconstr. Surg. Glob. Open.

[59] Persing S., Timberlake A., Madari S., Steinbacher D. (2018). Three-Dimensional Imaging in Rhinoplasty: A Comparison of the Simulated versus Actual Result. Aesthetic Plast. Surg..

[60] Topsakal O., Sawyer P., Akinci T.C., Topsakal E., Celikoyar M.M. (2024). Reliability and Agreement of Free Web-Based 3D Software for Computing Facial Area and Volume Measurements. BioMedInformatics.

[61] Lewyllie A., Cadenas De Llano-Pérula M., Verdonck A., Willems G. (2018). Three-Dimensional Imaging of Soft and Hard Facial Tissues in Patients with Craniofacial Syndromes: A Systematic Review of Methodological Quality. Dentomaxillofac Radiol..

[62] Todorović J., Zelić M., Jerkić L. (2022). Eating and Swallowing Disorders in Children with Cleft Lip and/or Palate. Acta Fac. Medicae Naissensis.

[63] Kosowski T., Weathers W., Wolfswinkel E., Ridgway E. (2013). Cleft Palate. Semin. Plast. Surg..

[64] Haleem A., Javaid M., Singh R.P., Rab S., Suman R., Kumar L., Khan I.H. (2022). Exploring the Potential of 3D Scanning in Industry 4.0: An Overview. Int. J. Cogn. Comput. Eng..

